# Patient Experiences and Prerequisites of Collaboration as Partners in Person‐Centred Care: An Interview Study

**DOI:** 10.1002/nop2.70133

**Published:** 2024-12-27

**Authors:** Lena Rosenlund, Sofie Jakobsson, Helen Lloyd, Anna Diffner, Åsa Lundgren‐Nilsson, Anna Dencker

**Affiliations:** ^1^ Institute of Health and Care Sciences, Sahlgrenska Academy University of Gothenburg Gothenburg Sweden; ^2^ Centre for Person‐Centred Care (GPCC) University of Gothenburg Gothenburg Sweden; ^3^ School of Psychology University of Plymouth Plymouth UK; ^4^ Department of Philosophy, Linguistics and Theory of Science University of Gothenburg Gothenburg Sweden; ^5^ Institute of Neuroscience and Physiology, Rehabilitation Medicine University of Gothenburg Gothenburg Sweden

**Keywords:** collaboration, communication, long‐term condition, patient experience, patient–professional partnership, person‐centred care, person‐centredness

## Abstract

**Aim:**

To explore what characterises communication and collaboration within a patient and professional partnership in outpatient care settings garnered from the experiences of persons living with long‐term conditions.

**Design:**

A qualitative descriptive study design.

**Methods:**

Semi‐structured individual interviews were conducted with 15 persons with long‐term condition/s who experienced outpatient treatment or follow‐up care. Data were explored through inductive thematic analysis. The COREQ checklist was followed.

**Results:**

The analysis revealed five themes: adapting and self‐managing in daily life, handling and carrying information, building trust and continuity, acting in a flexible and transparent dialogue and sharing the way forward. The participants described their personal and informal resources, and their actions to take control and manage health and well‐being. A person‐centred approach, sharing of knowledge and communication skills enabled the development of trust in the healthcare providers and their treatment and care. Communication was facilitated through availability, shared documentation, continuity and coordination of care. Collaboration was described as a flexible dialogue with mutual trust and transparency, shared learning and problem‐solving. Sharing the way forward was a process, alongside and important to the life‐changing process to cope with the illness.

**Conclusions:**

Prerequisites for the collaboration in outpatient settings were availability, continuity and a healthcare system that acknowledged, empowered and adapted to patients' health status, resources, everyday life and a patient's coping processes to manage their disease. For a co‐created, person‐centred outpatient care, it is important to acknowledge and/or collaborate with the patient's team of other healthcare providers and informal caregivers.

**Relevance to Clinical Practice:**

The study contributes to better understanding of patient preferences and prerequisites how to work in partnership and how to develop future services and person‐centred care for persons living with long‐term conditions.

**Patient and Public Contribution:**

Patients included in this study were participants during the data collection process.

## 1 Introduction

Demographic trends reveal that people are living longer and that the number of people ageing with chronic illness or multiple chronic conditions are increasing. This challenges the definition of health and how healthcare services are funded, managed and delivered worldwide (Krahn et al. 2021; World Health Organisation. 2018). Chronic or long‐term illnesses can be immediately life‐threatening, such as heart disease and cancer, while others develop over time and require intensive management, such as diabetes (World Health Organization. 2018). For the person, a chronic or long‐term illness means having to adopt improved health behaviours, the therapy used to treat the condition, ongoing vigilance, daily self‐management and coping with the disease both mentally and physically. It also means that the person will need to develop and maintain collaborations with the healthcare system and multiple healthcare providers across different settings (Bonino 2021).

## 2 Background

Person‐centred care (PCC) is recognised as an important aspect of healthcare quality and perceived as one way of achieving better outcomes for individuals with complex healthcare needs and to address the demands of the healthcare systems (Lloyd et al. 2017; Nolte, Merkur, and Anell 2020). PCC is based on the ethics of person‐centredness and an endeavour, with a holistic approach where the individual is not only seen as a patient but as a person and an active partner, and care is co‐created. The provision of person‐centred care requires a healthcare environment that fosters engagement and a positive therapeutic relationship, a partnership, between patients and their healthcare providers (Ekman, Ebrahimi, and Olaya Contreras 2021). PCC aims to support people to take control by developing the knowledge, skills and confidence they need to manage and make informed decisions about their own health and health care, which is crucial to people living with chronic conditions (Devan et al. 2018; Ekman, Ebrahimi, and Olaya Contreras 2021). PCC should also be integrated and tailored to the needs and preferences of the person and ensure that the person is treated with dignity, compassion and respect (Ekman, Ebrahimi, and Olaya Contreras 2021).

Caring for people with chronic diseases and with a person‐centred approach entails healthcare professionals to initiate and work in partnership with patient and their close ones, to enhance participation, health literacy and self‐efficacy to promote patients' self‐management, well‐being and coping with the disease in daily life over time (Devan et al. 2018; Farley 2020). PCC also requires that patients' and caregivers' experiences, knowledge and resources are better acknowledged and utilised in the healthcare system. To implement and practise PCC, it is important to learn from patients' experiences of how communication and collaboration between patients and healthcare staff is expressed in different contexts and over time.

## 3 Aim and Objective

The aim was to explore what characterises communication and collaboration within a patient and professional partnership in outpatient care settings garnered from the experiences of persons living with long‐term conditions.

## 4 Methods

### 4.1 Design

This study used a descriptive qualitative design with semi‐structured interviews as the data collection method, and an inductive thematic approach to analyse the data (Braun and Clarke 2022). The study is reported following the Consolidated Criteria for Reporting Qualitative Research (COREQ) checklist (Tong, Sainsbury, and Craig 2007).

### 4.2 Population and Recruitment

Eligible persons for this study were adults (> 18 years old) with experience of living with a long‐term or chronic condition and with an ongoing collaboration with at least one healthcare provider. Participants were recruited via voluntary response sampling. An invitation letter was sent by email to different Person Councils for patients and caregivers. Persons who voluntarily responded to the invitation letter were contacted by email by the first author who answered any questions and scheduled time for the interview together with the informant. All participants were informed, both verbally and by written information, about the study and its purpose. A total of 15 patients responded to the invitation and participated in the study, seven men and eight women, their ages ranged between 30 and 78 years with an average age of 59 years. Most of the participants (*n* = 14) had a diagnosis of cancer (breast cancer, lung cancer, prostate cancer, brain tumour, intestinal cancer, neuroendocrine cancer, chronic lymphocytic leukaemia and Ewing sarcoma) at different stages and had different prognoses. Two of the informants had two different types of cancer, one person also had multiple sclerosis, and another was under investigation for a joint and connective tissue disease. One person had Type I diabetes. The participants had lived with their chronic condition ranging from 7 months to 30 years and had an ongoing collaboration with at least one or more healthcare provider. Two persons also had experiences working as healthcare professionals.

### 4.3 Data Collection

Individual interviews were conducted April to June 2021 and each interview lasted 1–2 hours. Participants knew in advance that the study was conducted within a project focusing on person‐centred care and that this specific study focused on their communication and collaboration with the healthcare system. The interviewer used a semi‐structured interview guide. Participants were asked to elaborate on positive and negative experiences of care, collaboration and communication with their healthcare team during various stages of the care process, and how they planned, managed and documented their care themselves and/or together with the healthcare team. Probing questions were asked when necessary. The interviews started with a general open‐ended question to frame the persons perception of person‐centred care: ‘What is person‐centred care to you?’ The two first interviews were pilot interviews to test the interview guide. These interviews were included in the study and no adjustments in the interview guide was made. All interviews were performed by the first author (LR). The interviews were conducted via zoom due to the ongoing COVID‐19 pandemic. All the interviews were audio‐recorded to enable transcription.

### 4.4 Data Analysis

The interviews were transcribed in full and analysed according to Braun and Clarke's step‐by‐step guide to thematic analysis and with an inductive approach without predefined categories (Braun and Clarke 2022). The analysis followed a six‐step process: First, the interviews were transcribed verbatim and were read through with initial reflective notes to get familiar with the data, to get an overall sense of the content and to identify possible patterns. Second, two of the researchers (LR and ADi) operated a first general codification in NVivo 12 Pro software with an inductive approach, where meanings of phrases relevant to the aim of the study were identified and extracted in 1350 codes. To explicate their meaning, the codes were created from the semantic content of the data. Third, the codes were sorted into potential subthemes and themes driven by both the manifest and latent content in the interviews. Fourth, themes and subthemes were reviewed and revised. Thereafter, each theme was refined and defined to identify their essence, scope and content. Finally, the findings were summarised and quotations selected to provide evidence of theme credibility (Braun and Clarke 2022). Areas of alignment and disagreement were discussed during the whole data analysis process and the whole research team were involved in setting the themes. The first author (LR) use to work as a contact nurse for patients with brain tumours. LR practised reflexivity to consider how her prior experience may have shaped the interpretation of data through the process analysis.

### 4.5 Ethical Considerations

The study was carried out in accordance with the World Medical Association Declaration of Helsinki (Schmidt, Frewer, and Sprumont 2020), and the study was approved by the Swedish ethical review authority before any recruitment was initiated. Patients who were interested to participate in the study contacted the first author by mail. All participants received both written and oral information about the purpose of the study, the procedure, how the results would be presented, that their participation was on voluntary basis and that they could withdraw at any time. The recording of the interview started with the person gave oral consent to participate in the study and agreed to have the conversation recorded. All participants signed a consent form and sent back to the researcher by email or postal letter. Throughout the working process, data were handled with confidentially and only coded material was used in the process of analysis. Coded audio files, transcriptions and NVivo data were downloaded to a secure protected server at the University of Gothenburg only accessed by the research team and stored on the secure server for 10 years.

## 5 Findings

Five themes were identified from the interviews: adapting and self‐managing in daily life, handling and carrying information, building trust and continuity, acting in a flexible and transparent dialogue and sharing the way forward. An overview of the themes and selected quotations are presented in Table 1.

**TABLE 1:** Overview of the five themes with quotations.

Description	Quotes
Adapting and self‐managing in daily life	
This theme includes the person's actions as patient. The participants described their personal and informal resources (family, friends and peer patients) and how they adapted and self‐managed in daily life to take control and handle their health and well‐being	IP6: ‘I can't do much about having this diagnosis. It's a fact. It also a fact that I had to do operations and treatments. But I can do about everything else, how I live my life, if I exercise, what I eat, how I socialise, which fun things I do, if I am creative or do what I need to feel good and have a good quality of life. I think that was my motto during this whole time, that I can't have an effect on some things, but I can have an effect on myself. I think they (healthcare staff) could give different signals to patients. That the patient can have control over their life. I think that is important’. IP3: ‘I can say this, I was surprised how I reacted, I hadn't expected that I would react the way I did, I thought I would have crash‐landed and completely lost my footing. But I didn't. It was something completely different, I offered a supportive arm to myself and became my own best friend. Then I started taking charge: What do I need? What do I want? And then I made plans for that. So, I surprised myself. In the same way I thought I would want to read everything about breast cancer, but it wasn't like that, I didn't want to read anything. I just wanted to know what I needed to know, which was strange, but then you learned something about yourself’. IP5: ‘So there are maybe four patients sitting in a room and those of us who are breast cancer patients are sitting together. So, we are in the same boat and that means we talk to each other, and it is rather nice. We could encourage each other and give tips to each other’
Handling and carrying information	
This theme is about the patient as an information consumer and carrier, own documentations and how to handle and bring on information to more than one healthcare providers and to coordinate, navigate and communicate between those	IP1: ‘In the beginning of my illness I saved everything, appointment letters, information letters and I kept a kind of diary about how I was feeling and when I took these tablets… and so I had control over what I took… where I was in a chemotherapy cycle and so on. But as the years went by there weren't so many new things going on, nothing happening—everything was static, both treatment‐wise and how the illness had developed. And now I have all this… during the last three years I can see my electronic healthcare records. Now it doesn't feel as important to keep track. No, now I don't have my own documentation. There is not the same need any more either’. IP12: ‘It's just this with information, or that every specialist I meet only looks at it from their side, so I feel like I have to take responsibility as the bearer of information. But really the responsibility of bearing information should lie with the healthcare system to be person‐centred for me. Otherwise, it just becomes one organ at a time in some way. // So just to transfer the relevant knowledge. So that it can be used. Otherwise, it easily becomes a conflict when one tries to explain. For some doctors especially it becomes a conflict when one tries to explain to them. They say now it is this solution and when I ask but how does that work in relation to this? And then it can be difficult somehow’. IP10: ‘But I can actually feel, why didn't they do that then? Why do I need to ask for this? I shouldn't need to be the expert at rehabilitation. But actually, it is like that, you need to have the knowledge yourself to ask for help, is my experience’
Building trust and continuity	
This theme describes important aspects of communication with the healthcare system and included communication skills, building trust and a relationship, personnel characteristics of the healthcare providers, availability and continuity	IP6: ‘It depends a lot on how staff see me as a patient. They can see me as like them, a colleague, then we talk with each other like colleagues. Or they see me as a small child who doesn't know anything, who doesn't understand or know what's best for myself. So, I would say it 100% depends on attitude’. IP10: ‘And I was so happy because, I thought there and then, that he remembered! It's this type of thing that is exactly right, really! Yes, like when they see you. And to really know what is important for me, I think. That has been good’. IP9: ‘He (the doctor) has control and makes adjustments around my illness when it is needed. Then he is taking decisions and then you can obey! Without being submissive, so we work well together—that's how it feels’
Acting in a flexible and transparent dialogue	
In this theme collaboration was described as a respectful, flexible dialogue with mutual trust and transparency. The participants appreciated shared learning and problem‐solving. Different tools such as access to the electronic health records enabled the communication and collaboration	IP12: ‘And I have different needs, sometimes I want to talk on a level where I can find articles on it but sometimes, I don't want to do that at all, I just want to hand over everything to the healthcare system and shut off completely. Yes, so some way where you can be how you feel in those different times during the years. That is an important part’. IP13: ‘And then I got to learn how my leg works. // It has been excellent knowledge for me to get, to carry with me. And then she built my training programs around that and it has worked much better! I gained both understanding and that also matched my experience. And it allowed me to look at other things because I got this basic understanding. I got much better and above all I have got rid of those spasms’. IP11: ‘They see from blood samples straight away if there is anything wrong. But I never forget one time, on a Friday night, when I had got chemotherapy and a nurse called me and asked how I was. Well, thanks a little tired. Yes, I see that you are not feeling well but if you want to you can come here tomorrow and you will get two infusion bags of blood’
Sharing the way forward	
This theme includes the next step, care planning, coping and coaching by acknowledging and encouraging patients' own and informal resources, sharing expectations and values, own and common goals	IP6: ‘Above all it is my body. My journey, no‐one else's, so then it means working together. Again, try to have a joint goal, and who can do what to reach that goal. If one later calls it taking part or working together, yes, I think the most important thing is that you communicate that this is something we are going to do together’. IP12: ‘No, I am bad at having realistic goals. I am very bad at that. I have a load of goals, but they are not realistic really. I actually had a goal to finish my studies, but now I have got to the point that I am not even going to be able to do that and that grieves me. No, I could have done with help with ordinary goals, because those are still there with my healthy self in some way. I am quite good at accepting when I get worse but when I am good again, then I am terrible at having realistic goals’. IP14: ‘And that they actually hand over some parts to the patient and the patient treats themselves you could say. And that you do it with an awareness of this’

### 5.1 Adapting and Self‐Managing in Daily Life

Although the realisation of their illness came at different speeds for the participants, they described the diagnosis as a life‐changing event and several of the participants used the word ‘shock’. The participants described how they needed to adapt to the illness and its consequences, some lived with or had a risk of physical side effects due to treatment or the disease itself. Some described how they came to a point where they understood the disease was inevitably present and how they decided to focus on their health and well‐being. The adaption involved taking control by educating yourself to understand the disease and treatments. This by engaging in treatment decisions, in physical and/or mental rehabilitation and to make necessary changes in everyday life and roles, such as changing living habits and adjust to a healthy diet and lifestyle. Self‐managing the consequences of their illness involved how to be adherent to treatment, learn to identify and manage symptoms or side effects and determine when to contact healthcare professionals. Participants highlighted personal qualities that helped them in adapting, for example, being proactive and committed to problem‐solving. Some surprised themselves positively by becoming stronger or behaving differently beyond their own expectations. Doing something for yourself was described as literally a lifeline and a necessity to regain mental control and well‐being. This was not always acknowledged by healthcare providers and there were situations retold that healthcare providers told the patient that there was nothing they needed to do themselves.

Depending on one's network and preferences, the participants described their team as wider than health and social care services. Family, friends, workmates and peer support from fellow patients was an important resource for support in daily life. This included help to adapt to a healthy lifestyle, support in rehabilitation activities, information seeking and interpretation, social activities and giving encouragement and hope. This extended team also helped with practical things like transportation and household activities at times when the patient did not have the strength. Meeting other patients made them feel less alone in their situation and could inspire activities to cope with everyday life despite illness. Some information was even better to receive from a fellow patient and some participants suggested that health care could engage more in helping patients to meet a fellow patient. Some actively searched for a patient association, while some appreciated spontaneous meetings with other patients at the different healthcare settings. Other ways to involve with other patients were via social media or by reading other patients' stories portrayed in books. It was perceived good to have different contacts outside the healthcare system. On the other hand, when the healthcare system did not meet up to their needs and they instead got help outside the healthcare system could led them to questioning the healthcare system.

### 5.2 Handling and Carrying Information

At the time of diagnosis, information seeking was a priority to gain knowledge and to be able to engage in treatment decisions and self‐management. Some wanted only essential facts and others actively searched for information and wanted as much information as possible. The need for information did not just depend on personal characteristics, but also the situation, trust in the healthcare professional's expertise, health status and the process of coping with the disease. The participants had at the time for the interviews achieved a high level of knowledge about their health condition, treatment, coordination of care and how to manage their health and well‐being. To have this knowledge was considered powerful and a prerequisite to be able to participate in treatment decisions or to ask for specific interventions.

Most of the participants had their own documentation. The main reasons to document were to remember and learn, but it was also used to prepare for meetings, schedule activities and/or to keep track of health‐related issues, such as symptoms or side effects, in between encounters and over time. For some it was important to document what was said, and by who, in case of eventual mistreatment. Some of the participants also used different health applications to track their health or/and exercise. Other reasons to document were to keep a diary for the mental processing of having a disease. Reasons not to document were having access to shared documentation such as own's electronic health records, having a stable disease and/or preferring to focus on healthy thoughts.

In meetings with health care, there was a balance between being seen as engaged and active in a positive way such as educating yourself, being proactive and taking one's own initiative, and being seen as a troublesome patient. Patients often ended up in situations where they had to coordinate and act as an information carrier between different healthcare settings or between different authorities such as the Swedish Social Insurance Agency and their workplace. To be an information carrier could be strenuous, especially when it was not well received by the healthcare professionals. In order not to end up in such situations, participants described how they had left out information, such as being healthcare professional themselves, or certain knowledge they had to enable a discussion. Being an information carrier could also give a feeling that the professionals were not cooperating with each other, or fully prepared for the encounter, and it left the patient with the responsibility to both inform and coordinate. Some participants also expressed a feeling of having to fight for their own care or else nothing would happen. Being an information carrier to your close ones could also be difficult. Not everyone was open to talk about the disease and accompanying feelings, especially when they were not sure what is happening to themself. Some of the participants had experienced difficulties in telling family and friends about the disease, especially to their own children. Ways around this were to invite the family member/friend to participate in meetings with healthcare staff or to ask for support from the healthcare provider or a patient association.

### 5.3 Building Trust and Continuity

Initial meetings with healthcare professionals laid the foundations of trust and confidence. Participants described basic communication skills as important such as being seen, listened to and treated in a respectful and an empathetic way. They stated the importance of being seen as a whole person with own resources, wishes and needs and not just a diagnosis, a physical body or a patient. To be seen by healthcare staff, participants described the importance of conversational conventions such as introductions, recognition of the patient, taking time and making eye contact to initiate a trustful relationship. To be heard or listened to means, above all, not to be questioned but being taken seriously and getting serious responses to queries.

Participants described different personal qualities among the healthcare staff that they valued, for example, that the staff showed sincere engagement in their job, were updated, cared for the patients' interests, that they were being honest or straight forward, was accessible, and the ability to give hope. Building a trustful relationship was supported by transparent, understandable, individual information, shared learning and that the healthcare professionals acknowledged and remembered what the patient had said and what was important to them.

Having access to a known person, either a physician or a nurse, was highly valued but some patients also described that they did not need any personal access to a specific team member, they trusted a whole clinic as responsible for both availability and continuity. Continuity was more important at times when the patient felt ill or unwell. To be known by the healthcare provider meant that they did not have to tell their whole illness story from the beginning, less risk of being referred elsewhere, easier to get understanding and immediate help. Continuity or a personal relationship also gave a familiar feeling at appointments. When change in healthcare personnel occurred there was often a feeling of starting over. As needs and preferences changed over time, the healthcare personnel needed to be attentive and not to take them for granted.

Factors that hindered communication and building trust were the experience of not being heard, believed in or even being questioned by the healthcare professionals. It could also be when the healthcare professionals were unprepared in a meeting or directed focus towards someone else in the room. If the healthcare professionals talked over the patient in a hierarchical manner, or if they treated the patient as if they lacked understanding and took decisions without inviting the patient to be involved or ask questions. Communication could also be hindered if the healthcare professionals failed or forgot something like a referral, detecting a symptom or the sharing of important information and expectations. Other examples of failed communication were described as when healthcare professionals compared the patient to a group of patients with whom they did not identify. There were participants that described how they had overheard something that was for another patient, but thought it was for them. But most often, it was lack of information, common planning or mutual understanding that contributed to why participants felt misunderstood or even abandoned by healthcare providers. Another common experience to mistrust was when healthcare professionals did not communicate or coordinated with other professionals.

### 5.4 Acting in a Flexible and Transparent Dialogue

When talking about collaboration, several of the participants used the word dialogue. Participants valued transparent dialogue that could entail shared problem‐solving and learning by interpretation of different signs and symptoms, or a reflective dialogue where questions, thoughts and feelings were taken into account. It was important not only to build trust in the healthcare professional but also to trust the treatment and feel safe. The lack of a dialogue could lead to the patient changing healthcare professional.

For the participants, the availability and communication beyond the healthcare meetings was as important as the encounter. With complex needs, it was not always the general healthcare services that could help. Therefore, availability needed to be adapted to specific needs or disease and could vary from quickly needing a new prescription, to homecare or an emergency department.

Different tools enabled the communication and the collaboration. All participants had experience of the national ehealth services, where patients can digitally access and read their medical records. The participants stated that documentation in the digital medical records enabled transparency in the sense of being able to prepare and participate in discussions, compare one's own view to the healthcare professional's notes and having access to the individual care plan. Other tools enabling collaboration were different ways of communicating with the healthcare system such as email, chat or digital meetings. The participants found this positive due to the flexibility instead of waiting for office telephone hours or scheduled meetings. Participants had their own personal preferences on how to communicate with their healthcare providers, some preferred to make a phone call/talk while some found it easier to express themselves by text, email or postal letter. Some of the participants had experience of paper or electronically questionnaires where they could self‐report their queries and health. Even though they did not have much experience with questionnaires they were positive about using them in preparation of a meeting if healthcare personnel provided time to discuss the answers. Questionnaires were also mentioned as help to communicate delicate matters which otherwise might be missed and to document health and well‐being over time.

### 5.5 Sharing the Way Forward

Several of the participants had experienced different treatments, completed or ongoing. The next step of the treatment or follow‐up plan was known even though some participants pointed out uncertainty due to risk of recurrence. A care plan was most often used initially, or during periods of treatment or rehabilitation, however, it was seldom updated. Therefore, some of the participants lacked a plan during follow‐up periods and could miss being offered support. It was also unusual for the encounters to have a planned agenda.

Care planning or sharing the way forward was not just about making shared decisions or goals on a piece of paper, but also a mental process of coping with the disease. Sharing a common view was perceived as a process to visualise future needs and what to expect from treatment or rehabilitation. Participants stated the importance of individual, clear information about the pathway of the disease looking forward, chances of recovery, self‐management and if any preventable consequences of the disease, or treatment to avoid.

Some of the participants were used to setting goals for themselves, while others were not or chose not to set goals for fear of failure. Most of the participants had set goals related to everyday life, exercise and/or diet, which were not always shared with the healthcare professionals. Some of the participants wished for more support in defining realistic everyday goals. Some of the participants used the word ‘coach’ as something they appreciated or wanted to have. The coach could be either a family member or a healthcare professional. This coach could help with motivation and hope when needed. Teamworking with healthcare staff should include a respectful process of sharing common views and expectations regarding goals and treatment.

## 6 Discussion

In this study, the participants described both positive and negative experiences of communication and collaboration with healthcare professionals over time. They also described their resources and strategies living with long‐term or chronic illness. The results were presented in five main themes: adapting and self‐managing in daily life, handling and carrying information, building trust and continuity, acting in a flexible and transparent dialogue and sharing the way forward. Within these themes, key factors to patient experiences of person‐centred care and person‐centredness was confirmed important and further described for patients self‐managing their disease and collaborating with different healthcare providers over the care continuum. Starting with the patient's narrative and common rules of communication to trust in the healthcare professionals and the treatment, to a partnership with shared problem‐solving and learning through a flexible dialogue, to actions when needed, enhanced self‐efficacy, shared goals, hope and expectations.

The participants in this study highlighted the relational aspects of the partnership similar to the results by Wolf et al. (2017) investigating the partnership in hospital wards. In this study, the partnership or the collaboration was expressed as the importance of meeting someone whom they could trust and have a dialogue with, a dialogue which included transparency, shared learning and problem‐solving together over time. Without the prerequisites for an open and respectful dialogue, building trust and a partnership was not possible. Trust is central to positive healthcare relationships and is an essential component for engagement in PCC (Westlake et al. 2022). The participants also talked about trust when it came to treatment and treatment decisions. It was as important to understand and trust the competence of the healthcare team when it came to treatment, which has also been shown in other studies (Bailo et al. 2019; Zeh et al. 2021).

The participants gave several examples of positive and negative experiences of continuity or coordination of care where they expressed all four dimensions of continuity as described by Deeny et al. (2017). The participants used the word continuity mostly as personal continuity, the opportunity to build a trustful and therapeutic relationship with the same healthcare provider or clinic (interpersonal continuity) over time (longitudinal continuity). Also, the importance of transparency and the importance to share information in between healthcare settings, not leaving this to the patient alone, (informational continuity) was expressed. The shared documentation such as the electronic health records or care plans facilitated both information and coordination of care (management continuity).

Patients' preferences, abilities and experiences vary, and opportunities for patient participation in healthcare interactions needs to be flexible (Bonino 2021; Krahn et al. 2021). Health is dynamic and not static; it is a continuum, an adaption and a balancing act for the person (Krahn et al. 2021). The participants in this study described how their needs and preferences changed over time, from the time of diagnosis, during treatment periods to follow‐up care but also how their needs changed due to the disease status or other personal issues. There were times when they wanted to be more passive and taken care of, and there were times when they felt empowered and handled their disease themselves. Even though the person lives with their chronic illness 24 h a day, when the person seeks care, they can be the most vulnerable. For some of the participants, it was therefore important that the healthcare professionals were both attentive and flexible and did not take their previous preferences or abilities for granted.

There were also areas of improvement such as acknowledging the patient as a capable person and a competent patient. The narrative reveals important personal strategies and resources that should be acknowledged and considered (Ekman, Ebrahimi, and Olaya Contreras 2021). In this study, the participants described their journey and how they had adapted the disease to their daily lives. Self‐managing was important not just to fight the disease but to regain control of their own lives. This was not always acknowledged by healthcare providers. There where even situations retold where healthcare providers told the patient that there was nothing, they could do for themselves. This engenders learnt helplessness and patient passivity since personal resources were not acknowledged (Westlake et al. 2022). It also suggests that healthcare professionals fail to understand the importance of empowerment to support self‐management and self‐efficacy (Farley 2020; Westlake et al. 2022; Wolf et al. 2020). From the patient perspective, self‐efficacy and feeling of control can be both an outcome of the empowerment process and an enabling factor that they need in order to feel empowered, engage in self‐management and have an active role in their care process (Bailo et al. 2019; Bonino 2021; Farley 2020).

When it came to goals, most participants mentioned treatment goals such maintaining blood sugar levels. Some of the participants had their own goals, often around things that they could influence themselves such as exercise, or diet, or how they managed the disease mentally. Health‐related or wellbeing goals were not equally shared, discussed or documented. This was also confirmed in a study by Heckemann et al. (2022) where they looked at person‐centred documentation in patient accessible electronic health records. The content and language were mostly concerned with the documentation of symptoms and medical or care interventions. There was little person‐centred documentation of patients' preferences, wishes and needs, and shared decision‐making. Although not everyone felt the need to share their personal goals with their healthcare team, this could be an untapped source with the potential to empower the patient and to work in partnership (Bonino 2021; Rosenlund et al. 2021).

The participants described their team as wider than just healthcare services. Depending on one's network and preferences, important resources were the informal support, from family, friends and peer patients. Certain supporting roles around the patient could even be used interchangeable. For example, the coaching role around the patient, one person wanted a more coaching role from a healthcare professional while another person highlighted and had his wife as a coach. At times where one's own hope failed, it could be either a supportive healthcare professional, relative or fellow patient who could help. By acknowledging the patients' own resources, include informal care, and count them into the team may empower the patient and integrate formal and informal care. Then the patient may not feel abandoned by the healthcare system when they use support ‘outside’ the healthcare system. Also, informal caregivers needs to feel invited, involved and valued (Blanck et al. 2021). When it comes to persons living with chronic or long‐term condition, most care is undertaken by patients themselves and their families (Riggare et al. 2019). Therefore, a person‐centred team should be the patient's team and not just a team at the hospital. For a person‐centred outpatient care and teamwork, we may need to collaborate much more with other healthcare providers (inside and outside our own workplace) and informal caregivers around the patient (Blanck et al. 2021; World Health Organisation. 2018). This may also be part of the power shift, handing over the responsibility and confidence to the person.

In this study, the participants used and valued different eHealth services, especially the national e‐service (where the healthcare records are), but also other health applications. Studies have shown that patient‐accessible electronic health records have the possibility to contribute to more person‐centred care (Benjamins et al. 2021). Studies with cancer patients using different eHealth showed that the relationships with their healthcare professionals extended the boundaries of their care team. The psychological benefits of eHealth were feelings of safety, reassurance and a sense of control (Darley, Coughlan, and Furlong 2021). Also, the use of patient‐reported measures in clinical care to enhance person‐centred care seems to be underused. In a systematic review of Graupner et al. 2020, showed in most of the studies, that using feedback to patient and/or HCPs about the results from patient‐reported measures led to better symptom control, health‐related quality of life, patient satisfaction and patient–healthcare professional communication (Graupner et al. 2020). The use of patient‐reported measures may also contribute to shared documentation, where the patient adds information to the health records in a structured way. Implication for future studies could also be to further explore joint documentation.

### 6.1 Strengths and Limitations

The strengths of this study included the participants diversity in terms of gender, age and time since diagnosis. The participants also covered all different healthcare regions in Sweden, which were an advantage of conducting the interviews via zoom. Limitations was that some of the participants in the study had lived with their chronic condition for a long time and in some cases, they retold stories from the past, which may not resonate today. The participants were recruited via different patient councils and some of them may have been more engaged in their own and others care and represent a somewhat more experienced patient than the average. Another limitation is that most of the participants had experience of care due to physical illness, mostly various cancer diseases, which may not be representative for all chronic conditions, healthcare settings, community or social services.

## 7 Conclusion and Implications for Practice

In this study, key factors to patient experiences of person‐centred care and person‐centredness was confirmed important and further described for patients self‐managing their disease and collaborating with different healthcare professionals over the care continuum. Participants described what was important for them to participate and establish a partnership with healthcare providers, but also what was important to them to take control of to handle the challenges related to illness, treatments and care. Prerequisites to collaborate as partners were a person‐centred approach and communication skills such as being seen, listened to, believed in and treated in a respectful way both as a person and as a patient. Transparency and mutual problem‐solving through dialogues over time were perceived to underpin shared learning, decisions and a good collaboration. Prerequisites for the collaboration were also availability, continuity and a healthcare system that encouraged, acknowledged and adapted to patients' preferences, resources and preknowledge, invited and communicated with both formal and informal caregivers to empower the patient and to co‐create and integrate care.

This study contributes to a better understanding of preferences and prerequisites for collaboration and communication for persons living with long‐term or chronic conditions and information how to further develop future services and person‐centred outpatient care.

## Author Contributions

All authors contributed to the study design, conception and development of the manuscript. Lena Rosenlund performed the interviews, transcription of interviews and drafted the manuscript. Lena Rosenlund and Anna Diffner made the coding in NVivo. All authors were responsible for critical revision and finalising the themes and the manuscript. All authors have given final approval for the version to be published.

## Conflicts of Interest

The authors declare no conflicts of interest.

## Data Availability

The data that support the findings of this study are available on request from the corresponding author. The data are not publicly available due to restrictions e.g. their containing information that could compromise the privacy of research participants.
